# Soil Eating as a Psychological Coping Strategy for Women in Rural African Patriarchal Contexts

**DOI:** 10.3390/ijerph22060876

**Published:** 2025-05-31

**Authors:** Libopuoa Notsi, Mamochana Anacletta Ramatea

**Affiliations:** 1 Department of Anthropology and Development Studies, University of Zululand, KwaDlangezwa 3886, South Africa; notsil@unizulu.ac.za; 2 Department of Educational Psychology and Special Education, University of Zululand, KwaDlangezwa 3886, South Africa

**Keywords:** African patriarchy, African women, cultural practices, emotional relief, Lesotho, resilience, rurality, soil eating

## Abstract

In African patriarchal contexts, women have borne the dual responsibilities of mental and physical caregiving for their households and communities. These responsibilities often contribute to significant emotional, social, and economic burdens imposed by deeply entrenched gender and power structures. Alternative psychological coping mechanisms emerge to navigate these challenges, some deeply rooted in cultural and historical practices. One such practice is soil eating, which has been observed in various African communities. Historically linked to cultural and spiritual beliefs, soil eating has been largely unexplored from a psychological perspective. This paper examined soil eating as a coping strategy among women in Africa, investigating its role as a means of emotional relief, resistance, or a symbolic reclamation of agency in the face of oppression. Drawing on the indigenous knowledge systems (IKS) of eZiko siPheka siSophula as a psychological framework, this study engaged sixteen women aged 20 to 89 in Ha Makintane Lesotho to participate in focus group discussions and reveal their personal narratives, cultural traditions, and the intersections of gender, race, and class to understand how this practice helps them manage their mental and emotional toll of patriarchal dominance. This research contributes to discussions on resilience, survival, and the psychological strategies developed by marginalized communities, shedding light on the complex interplay between cultural practices, mental health, and gendered experiences of power.

## 1. Introduction

Cultural systems, such as patriarchy, have influenced societies and communities’ daily lifestyles, including those of women, for many centuries. These systems often establish power dynamics where men hold dominant positions in both the public and private sphere [1], while women are relegated to more subordinate roles [2]. In the rural African context, patriarchy manifests in various ways, such as through gendered divisions of labor, restricted access to resources, and limited opportunities for women’s education and participation in decision-making processes [1,3]. These entrenched power structures shape not only the physical but also the emotional and psychological realities of women, who often face considerable social and economic challenges [4].

The psychological impact of living in a patriarchal society can be profound, particularly for women who are expected to conform to strict gender norms and roles [5,6]. Feelings of powerlessness, frustration, and isolation can arise from a lack of agency and opportunities to express oneself freely [7]. These challenges often lead women to develop coping mechanisms to navigate the emotional toll of patriarchy. In some cases, these strategies may include behaviors that are considered unconventional or stigmatized within their communities [8]. One such coping mechanism, which has gained increasing attention in research, is soil eating, a practice known as geophagy [9,10].

In the context of this paper, soil eating refers to clay-eating, and has been observed across different cultures and religions, often linked to nutritional or medicinal beliefs. This is evident in a study conducted in Eastern Cape, South Africa, which indicates soil eating as a common practice amongst African pregnant women, and as an adaptive behavior to rectify nutritional deficiencies [11]. Similarly, in a study conducted in Nigeria, soil eating is practiced by pregnant women to satisfy cravings and alleviate gastrointestinal upsets and promote adequate bone formation in fetuses [12] (p. 20158). A study conducted in Kenya suggested that soil eating might be a cause of medical concern since it might contribute to intestinal worm infections [13]. However, in Tanzania, it was discovered that the risk associated with eating contaminated soil is determined by the element of interest, how much is consumed, and how often [14]. 

Although soil eating is perceived to have nutritional and medicinal value in the African context, its psychological functions remain less understood [15]. In rural African patriarchal settings, where women may experience multiple layers of oppression culturally and spiritually [16], soil eating could serve as an adaptive response to stress or emotional distress, and have a therapeutic purpose [9,15]. Research shows that this practice may provide a temporary sense of comfort or relief, acting as a form of self-medication in the absence of psychological resources or support systems [17]. It could be viewed as a means of reclaiming agency in an environment where women’s autonomy is often undermined [1].

Understanding soil eating as a psychological coping strategy requires an exploration of the broader socio-cultural context in which it occurs [18]. This practice does not merely exist in isolation; it is intricately connected to the intersection of gender, culture, and socio-economic realities in rural African patriarchal societies [19]. The practice of soil eating may offer important insights into the complex ways in which women in these communities negotiate their psychological well-being within systems that restrict their freedom and agency [20]. By examining this phenomenon, this paper aimed to contribute to a deeper understanding of the psychological coping strategies employed by women in patriarchal settings and the potential pathways toward holistic support for their psychological well-being.

The lack of psychological exploration and understanding of soil eating as a coping mechanism among women in African patriarchal contexts is central in this study. Despite the significant emotional, social, and economic burdens faced by women due to deep-rooted gender and power structures [18,19], there is limited research on how cultural practices, such as soil eating, may serve as a psychological strategy for emotional relief, resistance, or reclaiming agency. This gap in knowledge overlooks the potential significance of indigenous coping mechanisms rooted in cultural and historical contexts, particularly in relation to how they help women navigate the mental and emotional toll of patriarchal dominance [21]. This study sought to explore and understand the psychological role of soil eating, shedding light on its potential connection to resilience, survival, and gendered experiences of power.

This paper addressed the following questions:

1. Which factors contribute to the persistence of soil eating among women in rural African patriarchal contexts?
2. How does eating soil function as a psychological coping strategy for women in these contexts?

## 2. The eZiko siPheka siSophula Framework

Amongst theories of Indigenous Knowledge Systems (IKS) is eZiko siPheka siSophula (eZiko). The theoretical framework eZiko, which translates to “around the hearth, we cook and dish out” [18] (p. 5) can be interpreted as a metaphor for psychological resilience and coping strategies, which is proposed to create spaces for indigenous research [22] and emphasizes that cultural wisdom, practices, and beliefs are deeply embedded in the lived experiences and environments of indigenous communities. eZiko focuses on the traditional understanding of indigenous psychological health and coping mechanisms that are shaped by the environment, community, and ancestral practices [21]. It signifies a process of transformation and adaptation, where individuals, especially those who are marginalized, navigate the challenges they face within their socio-cultural realities [23].

This theory is rooted in the understanding of the multifarious interactions that the mind, body, and soul are interconnected with the land, the community, and the spiritual world [24]. This emphasizes the holistic approaches to psychological well-being through which people incorporate and embody social relations and cultural values [22]. This is strongly linked to the interrelatedness among girls, young women, and older women in Ha-Makintane who have long experienced the mental and emotional toll of patriarchal dominance. eZiko also offers the holistic philosophy of indigenous peoples and the ways of knowing that they have acquired over a long time [25].

The relevance of this framework to the present study lies in the way that it frames women’s coping mechanisms within their socio-cultural and environmental contexts. Soil eating, or “geophagy” [20] (p. 6) may be viewed as an indigenous coping strategy or behavior, one that ties back to the land and erudite ancestral practices of those who have gone before us [24], reflecting resilience within patriarchal structures that limit women’s agency. The psychological framework eZiko can provide insights into how women in rural African communities use this behavior to cope with stress, trauma, and socio-economic hardships [26]. In patriarchal contexts, where women are often marginalized [27], soil eating might represent an act of reclaiming control, emotional release, and survival [28]. Since this theory is perceived to advocate for culturally relevant and contextually appropriate efforts that draw on treasures and the wisdom of neglected knowledge and teachings of ancestors [29], it aligns with the aim of this study, which is to tap into the wisdom of neglected knowledge of African women in Ha-Makintane in Lesotho to reveal how they navigate through patriarchal power dynamics within their community.

## 3. Materials and Methods

### 3.1. Positionality

The principal researcher, who was conducting her doctoral thesis, which this paper is part of, brought a deep academic interest in understanding the intersections of gender, mental health, and cultural practices, particularly within the context of African communities. Her personal and scholarly journey allowed her to approach the study with a deep understanding of the socio-cultural factors impacting the lives of the participants. The authors’ focus on exploring alternative coping mechanisms, such as soil eating, was rooted in the awareness of the challenges and resilience exhibited by marginalized women in patriarchal societies. They have witnessed generational women in the community and neighborhood entangled in a matrix of patriarchal power that ignored women’s psychological needs. Their positionality in academia and women from the community under study provided an indigenous lens through which to examine the practice of soil eating. The researchers felt that it was appropriately befitting to bring some change to the geophagias, being women in Ha-Makintane who are labeled “bizarre” and misunderstood due to the practice [18] (p. 63). Soil eating can be an element of nutritional importance based on Abrahams and Parsons perspective that it may represent an attempt by an individual to restore the level of certain elements during times of nutritional stress.

### 3.2. Study Data

The identification of the study site was informed by the researchers’ childhood observations, which preceded the formal development of the research proposal. The researchers’ personal connection to the area played a significant role in selecting the study region, Ha Makintane, in Lesotho. Access to the study site was facilitated through the local traditional authority, which serves as the custodian of the community and ensures security and ethical oversight in research activities.

The study employed a qualitative approach, utilizing focus group discussions (FGDs) to explore the experiences of women who engage in the practice of soil eating. Eligible participants were adult women, aged 20 to 89 (see Table 1), who regard themselves as practitioners of soil eating. Purposive sampling, acknowledged as judgmental, selective, and subjective, was used to recruit the participants based on specific experiences of eating soil [30]. Participants were selected based on their residency in Ha Makintane for at least 10 years. Of the 16 women involved in the study, 9 were born and raised in Ha Makintane, while the remaining 7 were practicing soil eating before being married into the Makintane community, all from within Lesotho. The traditional authority of Ha Makintane played a key role in recruiting participants and assisting with data collection, particularly through the indigenous Lekhotla system, which is a form of community dialogue, akin to a talking circle [31], enabling focus group discussions to occur [32].

The focus group discussions were conducted in two phases. In the first phase, the principal researcher developed a focus group discussion guide, drawing on her years of observational experience in the community. This guide included a set of topic and probe questions to facilitate the exploration of participants’ personal experiences with soil eating, mental health, and the psychological impact of living under patriarchal structures [19]. Sensitive topics, such as soil eating, were approached with care, ensuring a safe and supportive environment for participants to share their experiences freely [33]. The researchers created a conducive environment that fostered open dialog to enable participants to comfortably discuss their situation [32], while also acknowledging that smaller groups might limit the depth of discussions on more general topics [34]. The researchers therefore ensured that the group size allowed for diverse perspectives while maintaining a manageable conversation flow.

The second phase of the focus groups included a demonstration segment, where participants volunteered to show how they engage in the practice of soil eating, including their digging techniques using their local tools such as sharp hard sticks and old iron nails or steel, while for collection, they used small enamel and plastic bowls. Prior to consumption, they apply hand-feel techniques to determine the texture of soil applicable to eat. This demonstration served to provide a more experiential understanding of the practice, adding depth to the data collection [35]. The focus group guide was originally formulated in Sesotho, the local language spoken by the participants, to ensure that all participants could engage fully with the discussion and then translated into English. Probing questions were used throughout the FGDs to elicit more detailed accounts of participants’ narratives, particularly those that addressed the intersection of gender, mental health, and the patriarchal system.

Recruitment started in December 2018 while the actual FDGs began in July 2019 and continued through December 2019. Participants were divided into two groups, each consisting of 8 women. The first group was selected based on those who had been eating soil for over 5 years or more while the second group were those who consumed soil rarely. A total of four FGDs were held for each group, providing ample opportunity for each participant to contribute. Sessions lasted between 1 to 2 h [36,37]. Data collection involved multiple methods such as voice recordings and field observations. These diverse data sources allowed for a comprehensive understanding of the practice of soil eating in the context of patriarchy and community life.

No financial incentives were provided to participants, but the study organizers shared a communal meal as a gesture of appreciation. Ethical clearance for the study was obtained, and informed consent was secured from all participants, with letters of support from the traditional authorities of Ha Makintane ensuring the ethical integrity of the research process [38].

### 3.3. Data Analysis

In this study, the researchers developed codes for all the focus group discussions (FGDs) to facilitate the analysis of the collected data. The narratives were transcribed and rigorously quality-checked to ensure accuracy [39]. The voice recordings of the Sesotho discussions were transcribed verbatim into Sesotho and then translated into English by a professional editor. A designated language quality checker reviewed the audio and transcripts to ensure linguistic consistency and resolve any discrepancies in the translation or conflicting narratives [40]. The researchers employed both inductive and deductive approaches using content analysis, with the software ATLAS.ti 8 aiding in narrowing down the emerging themes [41]. This approach allowed the researchers to systematically identify and refine the following key themes from the data: soil eating as a generational practice, as part of intergenerational conversations and a cornerstone for communication, as a coping mechanism to navigate power relationships, and as a coping mechanism toward femininity from the data, ensuring that both the context and the content of participants’ experiences were accurately represented [42].

## 4. Results and Discussions

### 4.1. Theme 1: Soil Eating as a Generational Practice

In addressing the research question, “Which factors contribute to the persistence of soil eating among women in rural African patriarchal contexts?” it was revealed from the findings in this study that the practice of soil eating emerged as a deeply rooted generational tradition, passed down through cultural and familial customs. Many participants reported learning the practice from their mothers or grandmothers and viewed it not only as an inherited coping mechanism but also as a vital connection to their heritage, identity, and ancestors. Quotes from some of the participants illustrate these viewpoints:

“*I started a long time ago, around the age of ten, with the girls I grew up with here in the village. It’s probably been more than thirty years, though I don’t eat soil every day. But I can’t go for a week without eating it. I learned it from our grandmothers, our aunties, and other girls and women from the village. It really helps us, especially when overthinking. During the process, I’ll get thirsty, and then I’ll stand up and drink some water*”

(P16)

“*I’m telling you; this is not a trivial matter. If it’s been happening for generations, it’s clear evidence that it’s a living tradition. Let me explain, our elders didn’t just do things for no reason. They acted with incredible foresight and wisdom, things that are beyond the understanding of those who take life for granted. That’s why we keep the practice of soil eating even today*”

(P11)

These quotes illustrate the generational transmission of the soil eating practice, highlighting how it was learned from older women in the community. It is evident that soil eating has been passed down from generation to generation, with participants commonly acknowledging that the practice has existed since ancient times. Like in other African communities, such as the Chagga women in Tanzania, where soil eating is considered sacred [43], this practice is often deeply embedded in cultural traditions. Among many African women, soil eating is particularly common during pregnancy, and in some cultures, it is a custom taught to children to ensure its continuation [44]. In the rural USA, soil eating was a generational practice until it began to decline due to the rise of modern social stigma [45].

This generational transmission of soil eating can be seen as an intergenerational heritage, functioning as a form of resistance and a means of reclaiming and restoring the historical trauma experienced by indigenous peoples. Culture is not simply taught but “caught” [46] (p. 48). In the case of P16, soil-eating is not just a cultural act but can be intergenerationally learned. The findings suggest that the practice of soil eating is more than a personal choice; it is a cultural heritage passed down through generations, carrying with it both historical and cultural practices.

### 4.2. Theme 2: Soil Eating as Part of Intergenerational Conversations and a Cornerstone for Communication

Another psychological factor contributing to the persistence of soil-eating rests in its function as part of intergenerational conversations, which plays a crucial role in empowering women. Participants reveal that these conversations help them foster communication skills and enable the sharing of strategies to navigate household and community dynamics. Their discussions on this issue include the following:

“*When we sit together and eat soil, older women teach us how to voice our thoughts respectfully, and that’s how we keep these traditions alive, but also learn how to live better with each other*”

(P13)

“*Yes! While sitting together, eating soil, we communicated all matters revolving around our lives as well as knowing the best strategies to use when expressing views within our communities.*”

(P2)

“*Talking with the younger girls and women in the village helps us a lot, especially when we discuss how to speak up at home and in the community, without fear*”

(P5)

Soil eating emerged as a key topic within intergenerational conversations among girls, young women, and adult women, serving as a cornerstone for communication in the community. These discussions not only provided insights into the practice of soil eating but also facilitated the sharing of strategies for expressing views and navigating power dynamics within households and communities. Evidence from the literature has proven that through these conversations, women can pass down knowledge and cautionary advice, and find ways to assert their voices, strengthening both individual and collective resilience [47]. These findings also align with the other scholars’ perspectives that soil eating embodies a culture of creative thought and human creation, reflecting the ingenuity and resilience of communities in their efforts to preserve practices that offer emotional, psychological, and social value [23]. It is therefore clear from these findings that soil eating becomes not only a personal practice but also a collective expression of cultural identity and survival.

### 4.3. Theme 3: Soil Eating as a Coping Mechanism to Navigate Power Relationships

During focus group discussions about “How does eating soil function as a psychological coping strategy?” Participants explain how they use soil eating as a vital coping mechanism to navigate stressful situations caused by unequal power relationships, particularly within their households and communities. The following extracts clarify:

“*When things get too tough at home, like when my views are ignored or I’m not allowed to speak freely, I go out and eat soil. It calms me down and helps me think more clearly*”

(P15)

“*Most of the time, we eat soil as a coping mechanism to manage challenging situations*”

(P1)

“*Yes!, Soil eating helps us to navigate challenges, and while we are together, we manage to build good relationships. We consider eating soil as a mode of peacebuilding within our community*”

(P8)

“*Since it is not easy being a woman in this community; we are often told what to do by men. But when we sit together as women and eat soil, most of the time we feel like regaining some of strength and power*”

(P7)

These responses suggest that soil eating serves as a form of coping mechanism, enabling women to regain a sense of control and some strength in environments where power dynamics often limit their voices. These findings are in alignment with eZiko principles that explain processes of transformation and adaptation, where individuals, particularly women who have been marginalized, adapt and gain resilience to navigate the challenges they face within patriarchal structures [19,26]. eZiko, which translates to “around the hearth, we cook and dish out” [22] (p. 5), is reflected in P7 and P8 perceptions and beliefs that when they sit together “being around the hearth” (fireplace), they start building strong relationships while eating soil, meaning as they “cook and dish out”, highlighting how they disseminate knowledge amongst themselves. The practice of soil eating thus becomes an essential tool for African women in Ha-Makintane to be resilient and manage stress caused by gendered power imbalances within their patriarchal context. These findings outline the principles of eZiko, which emphasizes the cultural wisdom deeply rooted in African knowledge systems to offer both a moment of support and a way to regain power and agency within unequal relationships [1]. It is indicative to note in these findings that the participants were aware of their cultural agency, which they reclaim through eating of soil to navigate power relationships within their communities.

### 4.4. Theme 4: Soil Eating as a Coping Mechanism Toward Femininity

Participants also discussed how they navigate the stressful pressures of femininity, particularly within the context of societal expectations and gender roles. Most participants expressed that the societal norms surrounding femininity such as the expectation to be quiet, obedient, and nurturing often create emotional strain and feelings of being constrained. Extracts below confirmed:

“*Sometimes being a woman in this community feels like a burden, where you are expected to be soft and not speak up. When I feel overwhelmed by these expectations, I turn to soil eating to relieve the pressure*”

(P4)

“*The pressure to always look and act a certain way can be exhausting. When I eat soil, it helps me momentarily escape those expectations and find some peace*”

(P6)

These reflections illustrate how soil eating serves as a coping strategy to manage the emotional and psychological burdens of conforming to societal standards of femininity. This aligns with the feminist and psychological literature, which confirms that women resort to different coping mechanisms to navigate their emotional and psychological burdens of societal expectations of femininity [48]. Though from a health perspective, the act of soil eating can be seen as impure [43], the findings in this study highlight the significance of how women who eat soil internalize societal pressures, leading them to seek coping mechanisms that offer temporary relief. This aligns with the idea of embodied coping strategies, where non-conventional behaviors serve to reclaim autonomy and regain control over one’s body and emotions [19]. The findings in this study argue that marginalized individuals, particularly women, often develop their own strategies to subvert dominant societal norms; therefore, soil eating in this context can be viewed as an act of reclaiming one’s agency in a world that continuously undermines women’s roles. Other participants confirmed:

“*When I eat soil, it’s like I’m reconnecting with something that belongs to me, something untouched by all the rules society has imposed on me. It’s a moment where I don’t have to be ’proper’ or fit into the box of what society expects from a woman. It’s my way of just being myself, even if it’s just for a moment*”

(P9)

“*I’ve always been told what it means to be a woman; how I should look, how I should behave. But when I eat soil, it feels like I’m breaking free from all those expectations. It’s a small rebellion that lets me feel like I’m not just a woman defined by others, but someone who has control over who I am*”

(P12)

“*Sometimes, society’s pressure to be perfect, whether it’s about my appearance or my behavior becomes overwhelming. Eating soil helps me feel grounded like I’m reconnecting with the earth, and for a moment, I can let go of all the things I’m supposed to be. It’s like a release like I’m allowing myself to just exist without the weight of everyone else’s standards*”

(P1)

The quotations reflect how soil eating can serve as a powerful coping mechanism for women seeking to reclaim their sense of self in a society that imposes rigid expectations of femininity. This resonates with several strands of feminists that highlight soil eating as an act that represents a moment of autonomy and self-reconnection [7]. The notion of soil eating as a “moment of autonomy and self-reconnection” aligns with the findings from the participants in this study, providing a brief but necessary escape from these pressures, and offering psychological release. This temporary escape allows women to affirm their individuality, a process of self-actualization [28]. For many participants, soil eating serves as both a subtle rebellion and a psychological release, allowing women to reconnect with their true selves, free from external definitions. Participants therefore consider this practice to nurture personal growth, affirm their individuality, and momentarily break free from the burdens of societal norms. Thus, this act enables women not only to challenge rigid gender norms but also to nurture personal growth, finding space for themselves outside the confines of society’s prescribed roles.

## 5. Conclusions

This study underscores the critical role that soil eating plays as a psychological coping strategy for women in Ha Makintane, Lesotho. Through the lens of the eZiko siPheka siSophula framework, soil eating emerges as a multifaceted and culturally significant practice. It intersects with key themes such as soil eating as a generational practice, as part of intergenerational conversations and a cornerstone for communication; as a coping mechanism to navigate power relationships; and as a coping mechanism toward femininity. More than just a physical act, it serves as a powerful coping mechanism that enables women to navigate complex power dynamics and societal expectations surrounding femininity, contributing to their personal resilience. Soil eating transcends its physicality to become a symbolic act of survival and resistance. It empowers women to confront patriarchal structures by fostering emotional survival and reinforcing connections among women. This practice is not only a tool for preserving psychological well-being but also a means of asserting identity and agency in restrictive environments. Through intergenerational wisdom, soil eating strengthens emotional resilience and provides a vital space for women to negotiate the pressures placed on them. Moreover, the practice embodies cultural preservation, allowing values to be passed down through generations while encouraging ongoing intergenerational communication. In the face of patriarchal power structures, soil eating functions as a quiet yet potent form of resistance, enabling women to assert their autonomy and create spaces for emotional expression and resilience.

## 6. Recommendations

Given the insights from the findings of this study, it is recommended that future research explore the broader implications of soil eating and other coping mechanisms in marginalized communities, particularly within patriarchal societies. Additionally, policymakers and mental health professionals should acknowledge the cultural and psychological pain underlying such practices and consider integrating them into culturally sensitive support frameworks. Further, intergenerational wisdom and communication should be prioritized in community-based mental health programs to empower women to cope with the challenges they face. This study suggests that fostering spaces for women to share and disseminate coping strategies can strengthen their emotional resilience and self-agency. By recognizing and respecting these cultural practices, societies can better support the psychological well-being of marginalized women, enabling them to navigate their environments with greater strength and autonomy.

## Figures and Tables

**Table 1:** Participants’ biographical information.

Participants	Age	Education Status	Marital Status	Employment Status
P1	20	Tertiary	Single	Unemployed
P2	20	Secondary	Married	Unemployed
P3	22	Primary	Married	Unemployed
P4	25	Tertiary	Single	Unemployed
P5	29	Tertiary	Single	Employed
P6	30	Tertiary	Married	Employed
P7	31	Secondary	Single	Self-employed
P8	32	Secondary	Widow	Unemployed
P9	33	Primary	Married	Unemployed
P10	36	Secondary	Married	Unemployed
P11	43	Primary	Married	Unemployed
P12	49	Secondary	Married	Unemployed
P13	56	Primary	Married	Unemployed
P14	68	Non-formal education	Married	Unemployed
P15	72	Non-formal education	Widow	Pensioner
P16	89	Primary	Widow	Pensioner

## Data Availability

The data supporting the results of this study are not publicly available due to privacy and ethical restrictions but can be accessed upon reasonable request from the corresponding author.
